# Non-Coding RNAs in Invadopodia: New Insights Into Cancer Metastasis

**DOI:** 10.3389/fonc.2021.681576

**Published:** 2021-07-05

**Authors:** Feiya Li, Burton B. Yang

**Affiliations:** ^1^ Division of Biological Sciences, Sunnybrook Research Institute, Toronto, ON, Canada; ^2^ Department of Laboratory Medicine and Pathobiology, University of Toronto, Toronto, ON, Canada

**Keywords:** invadopodia, noncoding RNA, ncRNA, cancer metastasis, circular RNA, circRNA

## Abstract

Invadopodia are actin-rich structures and their formation is implicated in cancer invasion and metastasis. Growing evidence has shown that noncoding RNAs (ncRNAs) play important roles in pathological conditions, including tumorigenesis and metastasis. Although this is still a new area of research, ncRNAs appear to be promising biomarkers and therapeutic targets for cancer metastasis. However, understanding the roles of ncRNAs in invadopodia is still in the early stages and far from clinical application. In this mini-review, we summarize the roles of ncRNAs in invadopodia functions and discuss them in a therapeutic context. The current challenges and gaps in this field are also raised, and we provide some open questions to facilitate new ideas in targeting invadopodia in anticancer therapy.

## Introduction

While invading into the dense extracellular matrix (ECM), the cell membrane must possess high levels of deformability (1). Such deformability is usually provided by cell cortex, a thin layer of actin mesh that typically underlies the cell membrane and have mechanical properties to create morphological change to form protrusions (2–4). Based on their physiological or pathological characteristics, these protrusions are classified into different groups including lamellipodia, filopodia, and invadopodia (5, 6). Invadopodia are found in cancer cells with high metastatic potential (7–9). They were first observed in 1980s in cultured chicken embryonic fibroblasts and were described as circular clusters (rosettes) with a different distribution of alpha-actinin and vinculin (10). In 1989, Chen et al. named these structures invadopodia to underlie their degradative abilities in cancer cells (11). Invadopodia are actin-rich structures that contain actin filaments and adhesion molecules. Extensive research has been conducted to characterize invadopodia and distinguish them from podosomes (3). There are specific molecules that are required for invadopodia formation and maturation. These include structural proteins (like cortactin, Tks4, and Tks5) (12, 13) and proteases, such as matrix metalloproteinases (MMPs) (14, 15). The formation leads to ECM degradation and thereby promotes cell locomotion and invasion (16, 17). Currently, therapeutic strategies targeting molecules that related to invadopodia formation show promise but are not yet satisfactory (18, 19). Molecular crosstalk is essential in every cellular process and the current state of invadopodia studies makes us consider that our knowledge on the regulatory network is far from complete. Increasing our understanding of the regulatory mechanisms might provide insights into the field of targeting invadopodia as an anticancer therapy. That is where non-coding RNAs (ncRNAs) step into the story.

Over 75% of human genomic DNA can be transcribed into RNA but only 2% of them encode protein (20, 21). In other words, more than 98% of RNA are “noncoding RNAs”. These noncoding RNAs have been underestimated for decades due to the limitation of solid detection and characterization methods, and the previous extensive scientific interest in the mRNA field. Proteins have always been regarded as the most important functional performers, but the regulation of protein function is a massively complex process. In the past decade, with the advantage of RNA technologies, ncRNAs have been broadly studied and their roles in serving as biomarkers or therapeutic targets in cancer have been extensively discussed (22, 23). In the following sections, we summarized recent research conducted in the field of ncRNAs in invadopodia involved in cancer metastasis, followed by the discussion of three major groups of ncRNAs: circular RNA (circRNA), long non-coding RNA (lncRNA), and microRNA (miRNA). The functions of these ncRNAs are summarized in **Table 1**.

**Table 1:** ncRNAs examples in invadopodia formation related cancer metastasis process.

ncRNA Type	ncRNA ID	Cancer Type	Invadopodia-related proteins	Function	Reference
**circRNA**	circSKA3	Human breast cancer	Tks5, Integrin beta1	Promotes tumor progression by complexing with Tks5 and integrin beta1, inducing invadopodium formation	(24)
**lncRNA**	MALAT1	Osteosarcoma	RhoA, ROCK1, ROCK2	Regulates cell migration	(25)
	MALAT1	Human breast cancer		Acts as competitive endogenous RNA (ceRAN), increases migration and invasion	(26)
	AFAP1-AS1	Nasopharyngeal carcinoma (NPC)	RhoA/Rac2 pathway	Loss of stress fiber formation	(27)
	MEG3	Thyroid carcinoma	Rac1	Negatively correlated to lymph node metastasis	(28)
	SchLAH	Hepatocellular carcinoma (HCC)	RhoA, Rac1	Inhibits cell migration	(29)
	ABHD11-AS1	Epithelial ovarian cancer (EOC)	RhoC, MMP	Induces tumorigenesis and progression of EOC	(30)
	SH3PXD2A-AS1	Ovarian cancer (OC)	Tks5	Related to overall survival (OS) of patients with OC	(31)
	SH3PXD2A-AS1	Colorectal cancer (CRC)	Tks5	Oncogenic in promoting cell progression	(32)
**miRNA**	miR-338-5p	Glioma	MMP2, ZEB1	suppresses glioma cell proliferation, migration, and invasion and accelerates its senescence and apoptosis	(33)
	miR-93-5p	Glioma	MMP2	inhibited proliferation and metastasis of glioma cells	(34)
	miRNA-10b	Glioblastoma multiforme (GBM)	MMP2, CTNNB1, RHOC	pleiotropically regulates invasion, antigenicity and apoptosis	(35)
	miR-145	Malignant glioma	MMP2, MMP9	ROCK1 serves as a novel target of miR-145 and positively regulate glioma cell invasion	(36)
	miR-222	Colorectal cancer (CRC)		overexpression promotes the migration and invasion	(37)
	miR-29a-3p, miR-200a	Colorectal cancer (CRC)	MMP2, ZEB1	AXT increases miR-29a-3p and miR-200a expression, represses the epithelial-mesenchymal transition (EMT)	(38)
	miR-204	Human breast cancer	Tks5, MMP2, MMP9	functions as tumour suppressor, directly inhibiting invadopodia extension and localized ECM remodeling	(39)
	miR-15b	Human breast cancer		Overexpressing MTSS1, a miR-15b target, decreased cell migration and invasiveness, decreased the formation of invadopodia and actin stress fibers, and increased the formation of cellular junctions.	(40)
	miR-612	hepatocellular carcinoma (HCC)		reduces invadopodia formation *via* HADHA-mediated cell membrane cholesterol alteration and accompanied with the inhibition of Wnt/beta-catenin regulated EMT occurrence	(41)
	miR-182	non-small cell lung cancer (NSCLC)	CTTN	Taget CTTN and generate tumor suppressing function	(42)
	miR-133a	esophageal squamous cell carcinoma (ESCC)	FSCN1, MMP14	Overexpression reduces FSCN1 and MMP14 mRNA protein expression. could serve as tumour suppressor of ESCC	(43)
	miR-16	human nasopharyngeal carcinoma (NPC)		Decrease of miR-16 upregulated FMNL1 expression in NPC	(44)

## Circular RNA (circRNA) in Invadopodia

Circular RNAs are a group of noncoding RNAs that possess a unique circular structure produced by a splicing process called “back splicing”. Such circular structures were discovered more than 4 decades ago but failed to stimulate interest until recently. Due to the advantages of RNA-sequencing techniques and the advancements of new computational pipelines specifically designed for circRNA analysis, increasing studies have screened out circRNA candidates that are potentially functional, and more experiments were designed to implicate the role of circRNA in different cellular events. Currently, most of the circular RNA studies have been focused on the cancer field. Although a lot of circRNAs have been studied and implicated in the process of cancer invasion, not many studies have taken specific look at the role of circRNA in the formation of invadopodia. This might be a current gap in the field. So far, there has only been one paper published specifically studying the role of circRNA in invadopodia formation and its implication in cancer metastasis.

Observed in microarray, circSKA3 was found significantly increased in breast cancer cell lines and human breast cancer tissues in our lab (24). In order to examine the function of circSKA3, an overexpression plasmid was constructed and the successful expression of circSKA3 was confirmed by northern blot and RT-qPCR. *In vitro*, overexpressing circSKA3 induced invasion. *In vivo*, circSKA3 increased tumor xenograft volume. Such effects were corroborated by the delivery of circSKA3 targeted siRNA. A morphological change was noticed in the stable transfected circSKA3 cell line, where podosome/invadopodia-like structures were obtained. Therefore, potential interactions of circSKA3 and invadopodia formation associated proteins were examined. A set of antibodies against invadopodia formation-related proteins (including Tks5, Itgab1, and MMPs) were used for immunoprecipitation to check the interaction with circSKA3. Tks5 and Itgab1 antibodies were able to pull down significantly higher levels of circSKA3 (24). A probe that specifically recognized the junction area of circSKA3 was designed and subjected to circular RNA pulldown assay. The pulldown results further confirmed the direct interaction between circSKA3, Tks5, and Itgab1. These interactions were abrogated when circSKA3 was silenced. Further evidence in supporting the association of circSKA3, Tks5, and Itgab1 was obtained from cell fractionation, co-localization in confocal microscopy, and blocking oligos that blocked binding sites between the circRNA and the proteins (24). These results demonstrated the interaction of circ-protein complex is important in cancer invasion through regulating invadopodia formation. Further studies in circRNAs in invadopodia formation-related cancer invasion are warranted. Such studies will represent potential target or strategies for novel cancer therapeutics.

## Long Non-Coding RNA (lncRNA) in Invadopodia

Long non-coding RNA (lncRNA) is another group of noncoding RNAs that usually possess a transcript longer than 200 nucleotides. Similar to circRNAs, lncRNAs have been implicated in playing vital roles in the process of tumor metastasis but not much has been done to illustrate their direct role in invadopodia formation. Currently, most of the lncRNAs implicated in invadopodia formation regulate Rho/ROCK signaling pathways, which are key signaling pathways involved in initiating cytoskeletal reorganization, including invadopodia formation.

The lncRNA MALAT1 increased the number of actin stress fibers in osteosarcoma cells and decreased the protein levels of RhoA, ROCK1 and ROCK2 and therefore regulated cell migration (25). Another study showed that MALAT1 acts as competitive endogenous RNA (ceRNA) with miR-1 and cdc42 in breast cancer cells, resulting in increased cell migration and invasion (26). LncRNA AFAP1-AS1 resulted in a loss of stress fiber formation in nasopharyngeal carcinoma (NPC) *via* affecting F-actin polymerization, as well as RhoA/Rac2 signaling pathway (27). Downregulation of lncRNA MEG3 was correlated with lymph node metastasis in primary thyroid cancer (28). MEG3 targeted Rac1 and reduced its protein expression, resulting in suppressed primary thyroid cancer migration and invasion. LncRNA SchLAH inhibited HCC cell migration through regulating RhoA and Rac1 (29). LncRNA ABHD11-AS1 induced expression of RhoC and MMP during tumorigenesis and progression of epithelial ovarian cancer (30).

Tks5 protein, encoded by SH3PXD2A gene, is a scaffolding protein essential for the formation of podosomes and invadopodia in untransformed cells. The lncRNA SH3PXD2A-AS1 was found to be related to overall survival (OS) of patients with ovarian cancer (31). Another study also reported the oncogenic role of SH3PXD2A-AS1 in colorectal cancer (CRC) by promoting cell progression (32). As a long noncoding counterpart of the linear Tks5 gene, the direct relation between SH3PXD2A-AS1 and invadopodia formation requires further investigation.

## MicroRNA (miRNA) in Invadopodia

miRNAs are evolutionally conserved 19–24 nucleotide long, single-stranded RNAs, initially transcribed from the genome as primary miRNAs and processed into precursor and mature forms through a biogenesis machinery including Drosha and Dicer. miRNAs have been shown to regulate mRNA through direct binding, or they can be sponged by circRNA to initiate regulatory actions. Compared to circRNA and lncRNA, more miRNAs have been investigated in the process of invadopodia formation involved in invasion. While some of them regulate invadopodia formation and metastasis through targeting invadopodia-related proteins, the others serve as downstream targets of other proteins to generate metastatic effects.

A couple of miRNAs have been implicated in the field of glioblastoma. miR-338-5p suppressed glioma cell invasion and other activities including proliferation and migration through decreasing FOXD1 expression and inhibiting the MAPK-signaling pathway activation (33). Higher expression of MMP2 and ZEB1 protein levels were found following miR-338-5p overexpression (33). miR-93-5p was found to inhibit metastasis of glioma cells through targeting MMP2 (34). miR-145 inhibited glioma cell invasion through targeting ROCK1 (36). Overexpression of miR-145 significantly reduced the invasive ability of U87 cells, and downregulation of MMP2 and MMP9 were found during this process as well (36). MiR-10b could regulate the orchestra of transcription factors and suppress invadopodia-related genes, including MMP2, CTNNB1 and RHOC (35). Overexpression of miR-10b decreased the invasive ability of mesenchymal type glioma cell U87-2M1 (35).

In colorectal cancer (CRC), miR-222 overexpression increased the migration and invasion of CRC cell lines (37). miR-29a-3p and miR-200a served as targets for Astaxanthin (AXT), one of the most common carotenoids (38). AXT increased miR-29a-3p and miR-200a expression levels and thereby suppressed the expression of invadopodia-related proteins MMP2 and ZEB1 (38).

In human breast cancer, miR-204 functioned as a tumor suppressor through downregulating Rab40b and Tks5 level (39). These Rab40b-Tks5- and miR-204-dependent pathway regulated MMP2 and MMP9 secretion as well, thereby affecting the ECM remodeling process. M5SS1 was found to be a miR-15b target, which decreased invadopodia formation and actin stress fibers when overexpressed in breast cancer cell lines (40).

In hepatocellular carcinoma (HCC), miR-612 decreased invadopodia formation through HADHA-mediated cell membrane cholesterol alteration (41). Moreover, patients with lower miR-612 expression levels but high HADHA expression levels showed a poor prognosis with decreased overall survival (OS). In non-small cell lung cancer (NSCLC), miR-182 was found to target invadopodia formation-related protein CTTN and generate a tumor suppressing function (42). In esophageal squamous cell carcinoma (ESCC), overexpression of miR-133a decreased invadopodia formation-related protein FSCN1 and MMP14 levels (43). The combination of FSCN1 and MMP14 expression was shown to be related to poor prognosis in ESCC patients. Patients with lower miR-133a levels exhibited poorer OS compared to those with higher levels. MiR-133a could potentially serve as a tumor suppressor in ESCC patients (43). The role of formin-like protein (FMNL1) was examined in human nasopharyngeal carcinoma (NPC) pathogenesis (44). Ectopic expression of FMNL1 largely induced cell invadopodia formation, and its depletion suppressed the invadopodia formation process. A downstream target, the metastasis-associated protein 1 (MTA1) was increased while miR-16 was decreased during overexpression of FMNL1. Thus, miR-16 provided a potential underlying mechanism for FMNL1 functions in invadopodia formation (44).

## Therapeutic Implications and Challenges

The broad interest in invadopodia is mainly focused on studying its role in cancer invasion and metastasis. Although invadopodia have a very complex structure, targeting its currently known main regulatory molecules have stimulated potential clinical interest. The involved molecules are expected to be possible anticancer therapies in the future. However, even though the current state of understanding of the major components of invadopodia seems promising, clinically targeting them has yet to be successful. This status quo deserves our attention since more complicated regulatory processes during invadopodia formation await detailed research. Such research includes the potential roles of ncRNAs in invadopodia formation.

There are couple of reasons to claim tremendous potential in the impact of ncRNAs in the field of invadopodia-related cancer invasion and metastasis. First, there are much more ncRNAs compared to mRNAs, making them capable of being the regulatory network of biological processes. Second, ncRNAs have characteristics that are beneficial in serving as biomarkers or therapeutic targets that mRNAs do not possess. For example, the unique circular form of circRNAs allow them to be stable in the microenvironment, making them ideally suited to be biomarkers. Third, the pluripotency of ncRNAs allow them to potentially serve as one target for different tumor pathogenesis.

However, considering ncRNAs for therapeutic strategies in the field of invadopodia-related cancer metastasis requires that we address some large gaps and challenges. The current study has not yet shown any invadopodia-specific ncRNA expression, which would be interesting and important to look at in order to understand the invadopodia formation mechanism better. Will there be any ncRNA that are exclusively expressed in the invadopodia? Or would there be some ncRNAs that are expressed closer to the inner cell membrane that have the potential to stimulate protrusions under normal physiological conditions? If there are, would the dysregulation of these ncRNAs manage the change in state from podosomes to invadopodia? Cell fractionation combined with RNA sequencing pipelines followed by *in situ* hybridization might provide new insights in answering these questions. The off-target effects should also be considered when discussing ncRNAs in a clinical manner. Pluripotency of ncRNAs might be implicated pros and cons at the same time. The pluripotency of ncRNAs allow them to potentially hold functions in many different tissues. Targeting the expression of a ncRNA as a therapeutic method might inadvertently cause pathological damage to other biological processes in another tissue type. Before targeting a specific ncRNA to treat invadopodia-related cancer metastasis, we need to understand its potential effects in the whole body to account for potential risks. Increasing the specificity of treatment and reducing potential off-target effects on other cells and tissues are needed in order for ncRNA-based therapeutics to become a viable treatment.

Despite the implications and challenges of examining the role of ncRNAs in a therapeutic manner, the field of invadopodia formation itself has critical issues that need to be resolved. First, even though actin assembly and adhesion molecules during the initiation process of invadopodia have been extensively studied, it is still unclear what, how, or why matrix degrading enzymes are recruited to the site of invadopodia to initiate their function. For example, although general inhibition of secreted MMPs decreased metastasis, eliminating metastasis may need new strategies. One such strategy could be to inhibit recruitment of the protease instead of blocking them. Second, it still remains to be uncovered how the components and the regulation of the components differ between invadopodia and other protrusions subtypes. Third, an off-target issue might also happen within the invadopodia components since they are actin-related cytoskeletal molecules, and it is known that they are involved in various cellular processes. How to specifically target invadopodia-related cancer metastasis without affecting other physiological processes needs further investigation. Finally, how to setup criteria for clinical trials and test molecule inhibitors in invadopodia research are also warranted. This does not only apply to the invadopodia field but also the whole metastasis field. Currently, the clinical trial system necessitates all anticancer drugs to present significant efficacy in reducing primary tumors in patients in phase II trials. Moving to phase III is not allowed until satisfactory efficacy was achieved. However, it is known that invadopodia mostly show functions in processes related to matrix degradation but not tumor proliferation. The standards in the current clinical trial system do not allow the effects of invadopodia inhibitors on metastasis to be adequately tested. Given the distinctive role of invadopodia in the process of cancer invasion and metastasis, potential drugs including invadopodia inhibitors should be tested in a better-designed clinical trial system or in a new set of standards of clinical trial evaluation parameters to examine their full potential in preventing metastasis.

## Discussion

Invadopodia formation is a very important step in the invasion process, and it is clear that the current research on this in the field of ncRNAs is insufficient, especially in the field of circRNA and lncRNA. On one hand, the traditional invadopodia field has largely not linked to the ncRNA field. The main therapeutic plan for this field is still a series of proteins that directly affect invadopodia formation, such as Tks5, MMP2, and MMP9. On the other hand, in the field of ncRNA, most studies only pay attention to invasion in general but do not study a specific process of invasion in detail. In this case, invadopodia formation may be an important process that has been overlooked. Interdisciplinary research is needed in the field of ncRNAs in invadopodia formation. This research may bring new ideas for clinical trials aimed at treating cancer metastasis.

It has long been challenging to characterize and visualize invadopodia formation *in vivo*. Lacking invadopodia-specific markers and filopodia-invadopodia distinguishing markers make it difficult to achieve characterization of invadopodia formation process in a 3D microenvironment. It is exciting to see that efforts have been made in characterizing invadopodia formation during intravasation and extravasation. Gligorijevic et al. was the first to record invadopodia and examined their roles in intravasation *in vivo* (45, 46). Later, the group further characterized the role of invadopodia *in vivo* and found that invadopodia degraded ECM surrounding blood vessels only in G1 phase of the cell cycle (47). Another *in vivo* model established by Dr. Hon Leong and his group successfully visualized the roles of invadopodia during extravasation (48, 49). These real-time 3D time-lapse intravital imaging systems are valuable for studying cancer metastasis, especially invadopodia formation-related metastasis process. They are also valuable for potential invadopodia inhibitor-based therapeutic agents screening.

It is gratifying that in the past decade, more and more scientific interest has been raised towards the field of noncoding RNA in cancer metastasis. Implications of ncRNAs in cancer metastasis are promising while the challenges in carrying them out as therapeutic targets are worth contemplating. The unique structure of circRNA, the lncRNAs targeting Rho/ROCK signaling, and the regulatory loop of circRNA/lncRNA/miRNA axis are all aspects that are worth initiating scientific discussion in terms of fully understanding invadopodia-related metastasis. How studying ncRNA properties can assist in exploring invadopodia formation-related metastastic process requires further mechanism research, and the pros and cons require our deep consideration.

## Author Contributions

FL and BY wrote the paper. All authors contributed to the article and approved the submitted version.

## Funding

Our studies were supported by grants from Canadian Institutes of Health Research to BY (PJT-153105, PJT-155962, and PJT-166107).

## Conflict of Interest

The authors declare that the research was conducted in the absence of any commercial or financial relationships that could be construed as a potential conflict of interest.
